# Emerging viral infections in immunocompromised patients: A great challenge to better define the role of immune response

**DOI:** 10.3389/fimmu.2023.1147871

**Published:** 2023-03-09

**Authors:** Chiara Agrati, Barbara Bartolini, Veronica Bordoni, Franco Locatelli, Maria Rosaria Capobianchi, Antonino Di Caro, Concetta Castilletti, Giuseppe Ippolito

**Affiliations:** ^1^ Oncoematologia e Officina Farmaceutica, Bambino Gesù Children's Hospital, IRCCS, Rome, Italy; ^2^ General Directorate for Research and Health Innovation, Italian Ministry of Health, Rome, Italy; ^3^ Department of Pediatrics, Catholic University of the Sacred Heart, Rome, Italy; ^4^ Department of Infectious, Tropical Diseases and Microbiology, IRCCS Sacro Cuore Don Calabria Hospital, Negrar di Valpolicella, Verona, Italy; ^5^ Unicamillus, International Medical University of Rome, Rome, Italy

**Keywords:** immune deficiency, immunopathogenesis, children, adult, persistent infection

## Abstract

The immune response to invading pathogens is characterized by the rapid establishment of a complex network of cellular interactions and soluble signals. The correct balancing of activating and regulating pathways and tissue-homing signals determines its effectiveness and persistence over time. Emerging viral pathogens have always represented a great challenge to the immune system and an often uncontrolled/imbalanced immune response has been described (e.g. cytokine storm, immune paralysis), contributing to the severity of the disease. Several immune biomarkers and cell subsets have been identified as major players in the cascade of events leading to severe diseases, highlighting the rationale for host-directed intervention strategy. There are millions of immunocompromised pediatric and adult patients worldwide (e.g. transplant recipients, hematologic patients, subjects with primary immune-deficiencies), experiencing an impaired immune reactivity, due to diseases and/or to the medical treatments. The reduced immune reactivity could have two paradoxical non-exclusive effects: a weak protective immunity on one hand, and a reduced contribution to immune-mediated pathogenetic processes on the other hand. In these sensitive contexts, the impact of emerging infections represents a still open issue to be explored with several challenges for immunologists, virologists, physicians and epidemiologists. In this review, we will address emerging infections in immunocompromised hosts, to summarize the available data concerning the immune response profile, its influence on the clinical presentation, the possible contribution of persistent viral shedding in generating new viral variants with improved immune escape features, and the key role of vaccination.

## 1 Introduction

In the last years, we continuously and suddenly faced with viruses that cause serious health concern. Immunocompromised (IC) subjects represent a heterogeneous group of pediatric and adult patients at increased risk of morbidity and mortality for infectious diseases compared to the general population, due to altered susceptibility to infectious agents and to impaired capacity to fight them, with possible critical clinical course and eventual long-term sequelae.

The reduced immune reactivity of IC patients may be due to the underlying disease itself (e.g., hematologic malignancy, solid tumors, congenital immunodeficiency, acquired immunodeficiency including AIDS with low CD4 counts, autoimmune disease, etc.) and/or to ongoing immunosuppressive treatments (e.g., hematopoietic stem cell transplantation (HSCT), solid organ transplantation (SOT), B-cell depleting drugs, steroids, etc.), which can strongly affect the immune system reactivity. Alongside these risk factors, IC patients have frequent contacts with the healthcare system that, in turn, may increase the risk of acquiring infectious diseases. In recent years, the pediatric and adult IC populations reached about 2-3% of the general population and the growing use of biological agents in several clinical settings will certainly contribute to increase the proportion of people showing some immunosuppressed traits.

An emerging infectious disease is one that either has appeared and affects a given population for the first time, or has existed previously, but is rapidly spreading, in terms of either number of people getting infected, or new geographical areas invaded. The majority of these infections are zoonotic, like SARS-CoV, West Nile Virus, Yellow Fever, and H1N1, and several factors (climatic, demographic, social) favour their spread (1). Other infections may re-emerge as a consequence of the failure in the healthcare system, as it occurred recently for measles (‘officially’ eradicated from the United States in 2000) and poliovirus, that reappeared in western countries afters years of silence (2, 3) possibly by new or previously underestimated way of transmission (4). In addition, already known viruses are recognized as potentially leading to important sequelae in vulnerable patients (e.g., Human Herpesviruses 6 and 7, or Adenoviruses) or cause of new diseases as a consequence of more potent immunosuppressive regimens recently available (e.g., polyomavirus BK). The list of rare and emerging viral pathogens in the immunocompromised population is quite long and unfortunately it is continuously growing, as demonstrated by recent relevant public health treats [Ebola 2013-2016, Zika 2015, Coronaviruses (MERS 2012, SARS-CoV-2 2019-ongoing), Influenza H1N1 (2009), H7N9 and Monkeypox]. Here, we aimed to summarize the evidences concerning: i) the complex scenario of the immune response to emerging viral infection in IC patients; ii) the impact of immune frailties on the clinical presentation of emerging viral infections and on viral shedding and iii) the vaccine efficacy in IC patients.

## 2 The immune response in IC patients: A complex scenario

Most viral infections cause self-limiting diseases of short duration. A well-coordinated humoral and cell-mediated immune response can effectively control and clear the infectious agent without causing damage to the host. However, the same virus in an IC host can result in more severe and longer disease, worsened by increased rates of bacterial and fungal superinfections (5, 6). The immune deficiency in IC patients can have different consequences, depending on the duration, the degree of immunosuppression and on the type of immune targets (humoral, cellular response or both).

One common feature in the pathogenesis of several emerging diseases (e.g. Flaviviruses, Filoviruses, SARS-CoV-2, etc.) is the role of early uncontrolled cytokine storm as the main driver of tissue damage, leading to immune-mediated multi-organ injury and disease progression (7–10). In this scenario, we could speculate that immunosuppression could paradoxically have a beneficial effect, dampening the excessive inflammatory response and reducing the immune-mediated damage (11). The huge number of SARS-CoV-2 infections in different clinical settings allowed scientists to study the impact of different immune frailties on the delicate balance between pathogenetic and protective actions during the course of a viral infection. The effect of immunosuppression induced by disease and/or treatment on COVID-19 clinical outcome can highlight a paradox: immunosuppressive conditions could be harmful in the initial phase of COVID-19, when the host immune response is necessary to inhibit viral replication. On the other hand, they might have a beneficial effect in the later, more severe, phase of COVID-19, preventing hyperinflammation and massive cytokine release that are, in fact, the main trigger of tissue injury and disease worsening (12). The observation that IC patients receiving biological drugs have a non-increased risk of disease severity (11, 13) suggests that the reduction of the immune reactivity might, indeed, contribute to dampen the cytokine storm-induced pathogenetic mechanisms. According to this hypothesis, the effectiveness of immunosuppressive treatment (e.g. dexamethasone, tocilizumab, baricitinib) has been tested in several randomised controlled trials, showing survival benefits in non-IC patients with SARS-CoV-2 infection (14–17). The detrimental role of inflammatory immune reactivity has been also confirmed in young children, whose tolerogenic immune profile has been associated with a generally asymptomatic/mild clinical course (18). Of note, the loss of this regulatory system can be responsible for the severe hyperinflammatory disorders occurring rarely in children with recent SARS-CoV-2 infection, such as MIS-C (Multisystem Inflammatory Syndrome Related in Children) (18, 19).

On the other hand, in more complex clinical settings (such as cancer patients and transplant recipients), the higher severity of SARS-CoV-2 infection could be explained better by the overall fragility, multiorgan involvement and chemotherapy-induced cytopenia inflammatory/suppressive profile rather than by impaired antiviral status (11, 20–23).

The tumor microenvironment (TME) is characterized by several cell types (e.g., fibroblasts, endothelial cells, macrophages, neutrophils, etc.) exerting potent immunomodulatory effects and contribute to cancer progression (24–26). The inflammatory/suppressive signals in TME promote the recruitment and expansion of several type of immunosuppressive cells such as myeloid derived suppressor cells (MDSCs), tumor-associated macrophages (TAMs) or Treg responsible for the strong inhibition of the host immune response (27–29). A similar suppressor cell expansion as well the increase in the neutrophil/lymphocyte ratio that characterize cancer patients (30) was also observed during viral emerging infection such as COVID-19 (31, 32). Their effects in dampening both antiviral and antitumoral immunity can therefore be exacerbated in cancer patients experiencing emerging infections.

A crucial role of T cells in protecting against the severe diseases has been defined in several emerging infections such as Zika (33), Dengue (34), SARS-CoV-2 (35) and influenza (36). The antiviral T cell effectiveness, together with their cross-recognition capability, give to these cells the role of crucial actor in IC patients with a deficit in humoral response, such as patients treated with B-cell depleting agents. Accordingly, CD8 T cells were associated with improved survival after SARS-CoV-2 infection in hematologic cancer patients showing an impaired humoral response, including those treated with anti-CD20 therapy (37). On the other hand, a loss of T cells can result in worsened morbidity and mortality after ZIKA infection (38). These results highlight the potential protective role of T cells in IC patients, and strengthen the beneficial approach of vaccination where available (39, 40) as well as of other cell-based approaches (41).

## 3 Immune failure, clinical outcome and persistent infection

The poor immune responsiveness in IC patients may reduce the inflammatory storm after infection, but, on the other hand, may impair the host’s ability to clear the virus, resulting in a persistent infection characterized by a prolonged viral replication at low level, in the absence of immune pathogenic processes. Common examples are represented by RNA viruses such as influenza A (42), rhinoviruses (43), and also polioviruses for whom chronic replication up to 28 years has been reported (44). In addition to these prominent examples, increasing evidences suggest that also emerging/re-emerging viruses can represent a relevant issue due to prolonged viral shedding in IC hosts.

A critical point in persistent infections is their association with enhanced viral genetic diversity with potentially higher rates of viral evolution compared to acute self-limiting infections, leading to the suggestions that IC hosts may represent an important reservoir for the emergence of novel viral variants possessing an immune-escape (45–47), or drug-resistant strain (48) phenotype. Among the emerging/re-emerging viruses, the most common persistent infections in IC patients have been observed with Dengue viruses, West Nile virus (WNV), SARS-CoV-2, hepatitis E virus, Molluscum contagiosum (49, 50), but also with new variants of adenoviruses (51).

In IC patients with a severe form of Dengue (e.g. shock syndrome or hemorrhagic fever), a prolonged period (median 30 days, range 18-80 days) of illness has been described (52, 53), and the full resolution of the infection coincided with the CD8 T-cell recovery, suggesting a role for cellular immunity in limiting the extent and duration of the infection (54). Main hematological dysfunctions of Dengue fever are leukopenia, thrombocytopenia and bone marrow impairment that can further worsen the chemotherapy-induced myelosuppression, resulting in bleeding and deep immune impairment that, in turn, is associated with an increased risk of secondary bacterial infections (55) and a high mortality rate (52, 56).

While WNV infection occurs asymptomatically in the vast majority of persons, the risk for neuroinvasive disease is significantly increased in IC patients (57). Moreover, IC patients may experience a severe neuropathological damage and multiorgan localization associated with prolonged infection (58). Of note, WNV can be detected in the blood for up to 28 days in IC patients, as compared to a maximum duration of 10 days in immunocompetent subjects (59).

Prolonged SARS-CoV-2 infections have been very well documented in IC patients. Among factors associated to this phenomenon, the B-cell depletion induced by a range of clinical or therapeutical conditions (e.g. hematological malignancies, bi-specific T-cell engagers (BiTEs), Chimeric Antigen Receptor (CAR)-modified T-cell therapy, HSCT or anti-CD20 monoclonal antibodies administration) represents a good example (60, 61). A few reported cases provide compelling evidence of prolonged infection even in SOT, AIDS, and/or other conditions (62). Nonetheless, several key questions remain about immunocompromised individuals with protracted COVID-19 not only to improve the well-being of the patient, but also to better understand intra-host viral evolution leading to the emergence of novel variants and to immune escape mechanisms (63).

The diagnosis of persistent hepatitis E virus infection has been rarely reported in IC patients, and the paucity of symptoms associated with persistent HEV infection strongly contributes to the delayed or missed diagnosis (64). Molluscum contagiosum virus is a double-stranded DNA virus component of the Poxviridae family, commonly seen in the pediatric population. It infects human keratinocytes resulting in small, umbilicated, flesh-coloured papules. Normally considered a self-limited disease, in IC patients, the presentation of MC is prolonged and can have different clinical presentation. Extensive and atypical presentation of normally self-limited infectious dermatosis has been reported in SOT recipients, patients with hematologic malignancies such as lymphoma and leukemia and in other iatrogenic immunosuppressive states (65). Moreover, it is well known that adenoviral infections can cause viral persistence and disseminated disease in IC patients. Recent observations rely to the potential role of new variants emerged both in United States and Asia. The primary source of disseminated infections in IC patients seems to be related to the reactivation of persistent ADV infection (66).

## 4 Vaccine efficacy: A critical point in immunocompromised patients

The impaired immune response in IC patients can make these patients more susceptible to infections, to develop severe diseases and, on the other hand, to be less responsive to vaccination. Influenza represents a good example: IC people can experience complicated disease characterized by progression to lower respiratory infection and bacterial superinfection, and, on the other hand, they show a poor response to vaccination (67). Indeed, the overall immunogenicity of influenza vaccine is lower in HSCT, where risk factors associated with a poor response include use of calcineurin inhibitors, chronic GvHD, shorter time post-transplant, as well as low IgM levels (68, 69). Accordingly, a lower immunogenicity has also been reported in SOT recipients as compared to the general population (70), and is dependent both on type of transplant and immunosuppressive regimen, being mycophenolate mofetil the drug with the greatest negative impact (71).

The number of IC patients at risk of encountering an emerging virus is growing both in endemic countries and in the context of tourist travel. Indeed, the advancement in the clinical and therapeutical management of IC hosts has promoted a significant improvement of their quality of life, which, in turn, has allowed them to travel abroad. The negative side of this improvement is the consequent risk to be exposed to tropical diseases, including rabies. Animal-associated injuries are common in travellers (72) and, in rabies enzootic countries, should lead to a prompt post-exposure prophylaxis (PEP). PEP regimen consists of the administration of a series of four rabies vaccine shots and rabies immunoglobulins (73) and results highly effective in preventing the disease in injured persons. IC patients usually receive a fifth vaccine dose, and should be tested for seroconversion 7 to 14 days following completion of the PEP regimen. Despite an additional vaccine dose, PEP seems to be less immunogenic in IC patients, as a detectable humoral response could be detected in a minority of tested patients (74). Nevertheless, further screening of immune response in IC patients is mandatory to define factors associated with inadequate response (75).

The incredibly successful development of highly effective SARS-CoV-2 vaccine platforms has changed the history of the COVID-19 pandemic, avoiding millions of deaths worldwide (76). In parallel with the success of the clinical standpoint, mass vaccination with mRNA vaccines has offered us a great opportunity to fully understand the immunological defects associated with different fragilities. The injection of the same antigen with the same schedule in millions of people with different ages, races, diseases, therapies, comorbidities, and the parallel analysis of both humoral and cellular immune response has opened a new scenario to fully define the mechanisms of immune-suppression and to design new host-directed immunological approaches.

A recent meta-analysis summarized that the seroconversion rate after two COVID-19 vaccine doses in IC patients was lower than that observed in healthy control: 92% (88–94%) for patients with solid cancer, 78% (69–95) for patients with immune-mediated inflammatory disorders, 64% (50–76) for patients with haematological neoplasia, and 27% (16–42) for transplant recipients (77). Accordingly, IC patients were at higher risk for symptomatic and severe COVID-19 (78) and account for more than 40% of hospitalised breakthrough cases, despite representing a much smaller proportion of the general population (79). In a prospective study including several of IC groups (hematological, solid tumors, neurological and rheumatologic patients) the independent predictors of seroconversion were age, diseases and treatment (39). In particular, the B cell-targeting therapy (e.g. anti-CD20 monoclonal antibody, anti-CD19 CAR T cells) was associated with the worst humoral response to vaccination, making these patients particularly vulnerable to SARS-CoV-2 infection. In contrast, spike-specific T-cell response is less susceptible to both diseases and treatment, highlighting its main role as a backbone of the immune response also in highly IC patients where they can exert a protective activities against the severe COVID-19 (35, 39, 80). The booster doses (third and fourth doses) improved the humoral response in all disease groups, although to a lesser extent in patients with hematological malignancies, whereas the T-cell response increased similarly in all IC groups (39, 81).

The analysis of the vaccine response in patients with transfusion-dependent thalassemia shed light on another aspect of immunity impairment. These patients were able to mount a good immune response to mRNA vaccination, with a strength similar to that observed in healthy donors, but their response decreased overtime faster than in healthy donors, highlighting a fragility in the persistence rather than in the power of the immune reactivity, which can be interpreted in light of an early immune senescence (82).

A huge effort has been making to define factors associated with a poor immunogenicity in different fragile patients with the awareness that these results could have a much greater impact than that of the COVID-19 vaccination context. The definition of the molecular mechanisms of immune fragility linked to individual pathologies, therapies, comorbidities, age, etc., can indeed provide new useful tools for improving the management of these patients and for designing the best vaccination strategy. Several critical issues need to be pointed out when addressing the vaccination in the fragile clinical settings, such as the best time for the vaccine administration (e.g. time from transplantation, time from anti-CD20 therapy start, etc.), the possibility to temporarily discontinue the immunosuppressive treatment and the definition of the optimal vaccine schedule.

Finally, in the development of new vaccination strategies against emerging and re-emerging infectious diseases, we need to remind that IC patients cannot receive live vaccines because their immune suppression condition may lead to the uncontrolled replication of the vaccine virus, with consequent trigger of virus-induced pathogenic events.

## 5 Conclusion

Fragile patients are characterized by different immune impairment signals affecting the overall immune protection that can be further worsened by emerging viral infections. Accordingly, the management of these infections in IC patients represents a relevant clinical challenge.

**Figure 1:**
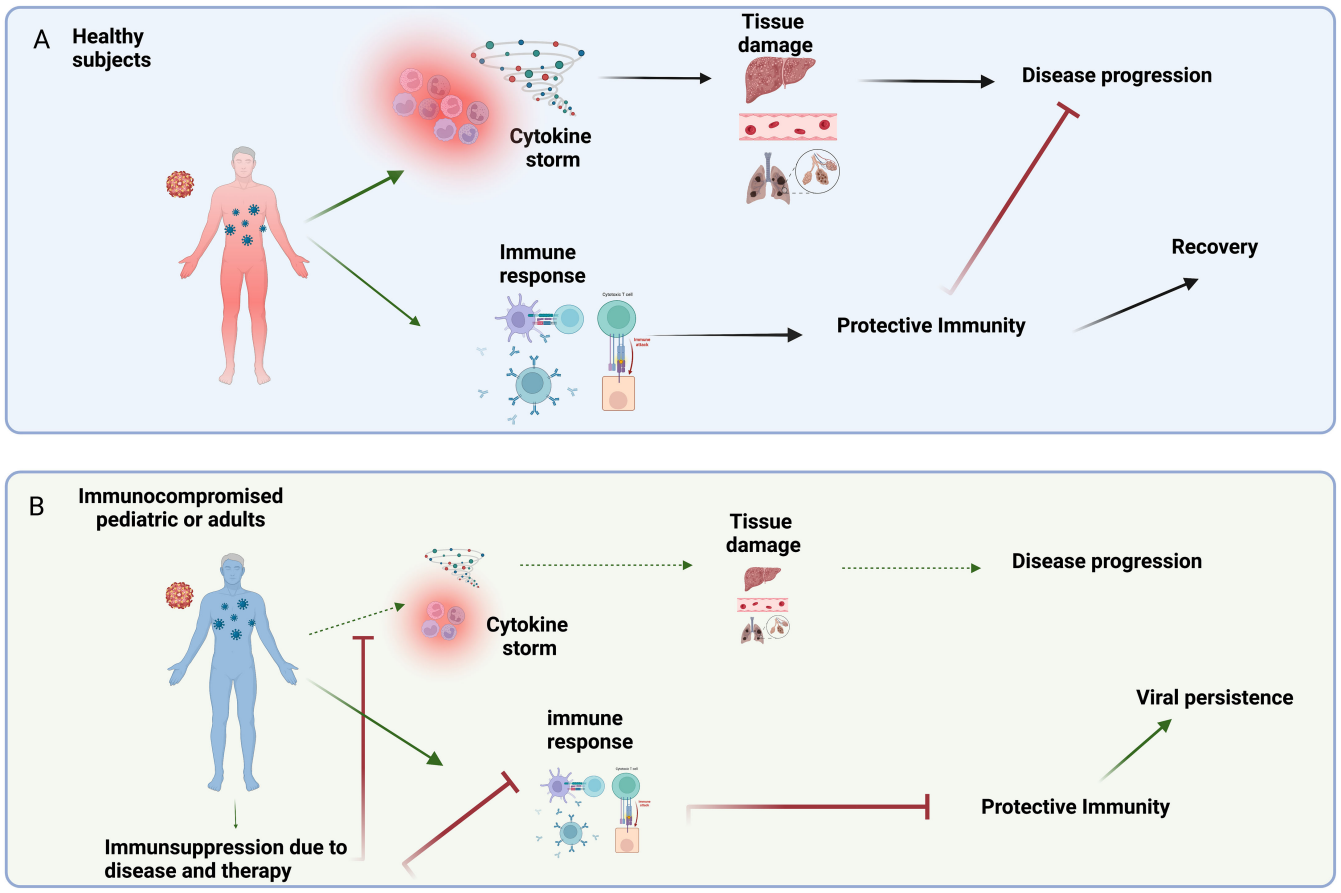
**(A)** In healthy subjects, emerging viral infections induce an uncontrolled/imbalanced release of pro-inflammatory mediators (e.g. cytokine storm) that can contribute to tissue damage and disease progression. On the other hand, the competent immune system can mediate a protective immune response leading to clinical recovery. **(B)** The low immune reactivity characterizing the IC patients, significantly reduces the inflammatory response partially avoiding tissue damage and disease progression. On the other hand, the immunosuppression due to disease and/or therapy can reduce the ability to induce a protective immunity, thus resulting in persistent non-severe infections. Created in BioRender.com.bio.

In healthy subjects, emerging infection may induce an excessive innate inflammatory response (cytokine storm), representing the main diver of tissue injury and disease progression. At the same time, the immunocompetent immune system recognizes viral antigens, differentiates, and mediates a protective immune response, resulting in virus clearance and prompt clinical recovery (**Figure 1A**). On the contrary, in the IC individual, we can observe a paradox: the low immune reactivity significantly reduces the inflammatory response partially avoiding tissue damage; on the other hand, the immune impairment can be unable to effectively clear the virus, resulting in persistent non-severe infections (**Figure 1B**). In this perspective, immunosuppression might be non-detrimental or even advantageous, as demonstrated in several clinical trial addressing SARS-CoV-2 infection.

## Author contributions

CA, FL, AC, MC, GI conceived the review; CA, BB, CC and VB drafted the paper; CA and VB prepared the figure; FL, AC and GI reviewed the paper; all authors agreed on the final version.
